# Neutrophil Extracellular Traps in Pulmonary Diseases: Too Much of a Good Thing?

**DOI:** 10.3389/fimmu.2016.00311

**Published:** 2016-08-15

**Authors:** Bárbara Nery Porto, Renato Tetelbom Stein

**Affiliations:** ^1^Laboratory of Clinical and Experimental Immunology, Infant Center, Institute of Biomedical Research, Pontifical Catholic University of Rio Grande do Sul, Porto Alegre, Brazil; ^2^Laboratory of Pediatric Respirology, Infant Center, Institute of Biomedical Research, Pontifical Catholic University of Rio Grande do Sul, Porto Alegre, Brazil

**Keywords:** neutrophil, neutrophil extracellular traps, NETs, pulmonary diseases, lung infection, respiratory infection, bacteria, viruses

## Abstract

Neutrophil extracellular traps (NETs) arise from the release of granular and nuclear contents of neutrophils in the extracellular space in response to different classes of microorganisms, soluble factors, and host molecules. NETs are composed by decondensed chromatin fibers coated with antimicrobial granular and cytoplasmic proteins, such as myeloperoxidase, neutrophil elastase (NE), and α-defensins. Besides being expressed on NET fibers, NE and MPO also regulate NET formation. Furthermore, histone deimination by peptidylarginine deiminase 4 (PAD4) is a central step to NET formation. NET formation has been widely demonstrated to be an effective mechanism to fight against invading microorganisms, as deficiency in NET release or dismantling NET backbone by bacterial DNases renders the host susceptible to infections. Therefore, the primary role of NETs is to prevent microbial dissemination, avoiding overwhelming infections. However, an excess of NET formation has a dark side. The pathogenic role of NETs has been described for many human diseases, infectious and non-infectious. The detrimental effect of excessive NET release is particularly important to lung diseases, because NETs can expand more easily in the pulmonary alveoli, causing lung injury. Moreover, NETs and its associated molecules are able to directly induce epithelial and endothelial cell death. In this regard, massive NET formation has been reported in several pulmonary diseases, including asthma, chronic obstructive pulmonary disease, cystic fibrosis, respiratory syncytial virus bronchiolitis, influenza, bacterial pneumonia, and tuberculosis, among others. Thus, NET formation must be tightly regulated in order to avoid NET-mediated tissue damage. Recent development of therapies targeting NETs in pulmonary diseases includes DNA disintegration with recombinant human DNase, neutralization of NET proteins, with anti-histone antibodies and protease inhibitors. In this review, we summarize the recent knowledge on the pathophysiological role of NETs in pulmonary diseases as well as some experimental and clinical approaches to modulate their detrimental effects.

## Introduction

Neutrophils are key players in microbial killing, being the first immune cells to achieve the site of injury or infection (1). Therefore, neutrophils act as the first line of defense against microorganisms through phagocytosis, release of reactive oxygen species (ROS), and degranulation (2). Aside from these traditional mechanisms, neutrophils are also able to extrude DNA lattices, called neutrophil extracellular traps (NETs), which entrap and facilitate the killing of bacteria, fungi, protozoa, and even viruses (3–8). NETs are composed of decondensed chromatin fibers coated with antimicrobial proteins, such as histones, neutrophil elastase (NE), myeloperoxidase (MPO), and α-defensins (3, 7). Besides being expressed on NET fibers, NE and MPO also regulate NET formation (9). Differently, the participation of NADPH oxidase-derived ROS in NET release seems to be a matter of time of stimulation. While ROS are required to NET generation in time points beyond 1 h after stimulation (10, 11), a very rapid process (5–30 min) of NET extrusion has been reported to be ROS-independent in response to *Staphylococcus aureus* and *Candida albicans* (12, 13). Furthermore, histone deimination by peptidylarginine deiminase 4 (PAD4) is a central step to NET formation (14). Additionally, the release of these DNA threads requires autophagy and activation of p38 MAPK and the Raf-MEK-ERK signaling pathways (15–17). However, it is important to keep in mind that the specific cell components and signaling cascades may vary depending on the stimulus (18).

The primary role of NETs is to prevent microbial dissemination because of its stringy structure, and to kill pathogens due to the high local concentrations of antimicrobial molecules (19). However, these attributes make NETs potentially detrimental to the host. The pathogenic role of NETs has been described for many human diseases, infectious and non-infectious (20), being particularly important to lung diseases. Netting neutrophils in the lung tissue are able to disturb microcirculation and NETs produced in the pulmonary alveoli can expand easily, filling the lungs, as is the case for cystic fibrosis (CF) (19, 21). Therefore, NET formation must be tightly regulated. In this review, we summarize the recent knowledge on the pathophysiological role of NETs in pulmonary diseases as well as some experimental and clinical approaches to modulate their detrimental effects.

## Cystic Fibrosis

Cystic fibrosis is a fatal hereditary disorder resulting from mutations in the CF transmembrane conductance regulator (CFTR) anion channel (22). This anion channel is responsible for the transport of chloride ions across the epithelial layer of the airways, which is necessary for the production of thin, freely flowing mucus. Therefore, the lungs of CF patients produce large amounts of thick mucus, leading to an obstruction of the airways and colonization by bacteria (23). Typically, CF infants are rapidly colonized by *Haemophilus influenzae* or *S. aureus*, or both. Over time, *Pseudomonas aeruginosa* represents the main bacterial pathogen infecting CF lungs (23, 24). Due to these frequent infections, there is a massive neutrophil infiltration to the lungs and development of chronic inflammation (25, 26). The chronic and progressive lung disease accounts for morbidity and mortality of CF patients (25).

Cystic fibrosis sputum constituents include DNA, NE, MPO, and other neutrophil proteins (27), as it has been shown that bronchoalveolar lavage fluid (BAL) from CF infants presented high concentrations of DNA, which correlated with neutrophil numbers in BAL (28). However, the great amounts of extracellular DNA in CF sputum were considered to be from necrotic neutrophils and lung tissues (29). More recently, several studies have demonstrated that NETs and NET-associated proteins are present in CF sputum (30–35). Marcos and coworkers quantified free DNA levels in airway fluid from CF patients and found that those patients with poor pulmonary function presented higher levels of extracellular DNA compared to patients with mild lung disease (36), indicating that the accumulation of NET–DNA in the airways contributes to airflow obstruction in CF. Moreover, analysis of CF sputum samples revealed that elevated levels of macrophage migration inhibitory factor (MIF), a potent pro-inflammatory cytokine, correlated with poor pulmonary function, and MIF was able to induce NET formation (33). Although many of the microorganisms that colonize CF airways have been shown to induce NET formation directly (4, 6, 12, 37, 38), pro-inflammatory cytokines and neutrophil chemokines present in CF lungs are also able to stimulate NET release (30, 33), thus perpetuating the inflammation.

Neutrophil recruitment and NET production in the lungs would be key events to fight against invading microorganisms, but their mission accomplishment is profoundly compromised in CF airways as patients often suffer chronic infections. Together with the failure in killing the bacteria, the excessive release of extracellular DNA accounts for biofilm formation by *P. aeruginosa*, and NETs act as a proinflammatory component of biofilms (39). Furthermore, over the time of infection in CF airways, *P. aeruginosa* is able to acquire resistance to NET-mediated killing (38), probably due to its hypermutability, a well-described mechanism for *P. aeruginosa* adaptation within CF lungs (40–42). In addition, it has been recently demonstrated that sub-inhibitory concentrations of LL-37, a NET component, triggers *P. aeruginosa* mutagenesis in chronic infections (43). Interestingly, *P. aeruginosa* triggers the release of the eicosanoid hepoxilin A3 by lung epithelial cells, which induces neutrophil transepithelial migration and is a natural inducer of NET formation (44, 45). Thus, the excessive release of NETs coated with proteases, together with the colonizing bacteria may worsen pulmonary inflammation and dysfunction. Besides NETs being able to directly induce endothelial and epithelial cell death *in vitro* through histones (46), MPO and NE expressed on NET fibers could exacerbate lung pathology through the destruction of connective tissue and degradation of endothelial cell matrix heparan sulfate proteoglycan (47, 48). Moreover, it has been shown that NE cleaves host proteins at the site of inflammation (49). Additionally, histones are highly cytotoxic to endothelial cells *in vitro* and are lethal in mice (50). Altogether, these findings highlight the need to target the massive NET release in CF.

The current therapy to improve CF symptoms is the administration of recombinant human DNase I (pulmozyme/dornase alpha) (51). DNase inhalation is one of the successful treatments for CF, as it improves lung function and reduces infectious exacerbations (52); however, it is not effective for all CF patients. Therefore, alternative therapeutic options are desired. Dubois and colleagues have demonstrated that DNase administration to CF sputum dramatically increased its elastase activity (53). Thus, the combined administration of DNase and an elastase inhibitor could be useful to avoid the devastating effects of excessive proteases in CF lungs. There are also candidate drugs to inhibit NET release, such as chloroquine and PAD4 inhibitors, however neither of these molecules has been evaluated in animal models of CF.

## Asthma

Asthma is a chronic heterogeneous inflammatory disorder of the airways characterized by airway inflammation and reversible airflow obstruction (54–56). Asthmatic subjects present periods of stable condition that alternate with severe episodes of exacerbations, leading to the impairment of lung function (57). Asthma symptoms include recurrent wheezing, coughing, and shortness of breath (55). This very complex disease is caused by multiple environmental factors that act in combination with hundreds of susceptibility genes (55). Asthma has been seen for a long time as an eosinophilic disease (56); however, in recent years, it has become evident that some asthmatics have a prominent neutrophilic inflammation in the lungs (58). Patients with neutrophilic asthma usually present a severe form of the disease that does not respond to the classical treatment with glucocorticoids (59, 60). In addition, glucocorticoid administration to neutrophilic asthmatics could aggravate lung inflammation, since glucocorticoids can prolong neutrophil survival (61). It has been described that neutrophils recruited to the lungs of atopic asthmatic patients generated NETs colocalized with elastase (62). In some patients, the number of neutrophils and NET-releasing neutrophils exceeded the number of eosinophils in the lungs. In this study, Dworski and colleagues also demonstrated that eosinophils infiltrating the airways of atopic asthmatics were able to release eosinophil extracellular traps (EETs), which colocalized with eosinophil granule proteins, such as major basic protein (MBP) and eosinophil cationic protein (ECP). Similar to the first study reporting the release of EETs from viable eosinophils (63), the DNA actively released by eosinophils in asthmatic lungs was from mitochondrial origin, and not nuclear (62). Interestingly, allergen challenge did not increase EET or NET formation in the airways of asthmatic subjects (62). Thus, what would be the role of EETs and/or NETs in the pathogenesis of asthma? And what would be the cause of EET/NET release in asthma? Taking into consideration the high concentrations of proteases anchored in extracellular DNA traps, one can assume that these enzymes could contribute to epithelial and endothelial cell damage, a hallmark of asthma. On the other hand, the formation of DNA lattices could protect the host against possible infections secondary to cell damage. Currently, these and many other questions regarding DNA traps formation in allergic diseases are still open for debate. More recently, it has been demonstrated that eosinophils from asthmatic mice release EETs decorated with eosinophil peroxidase (EPO) with no signs of cell death (64), indicating that DNA release is an active process. In addition, recombinant human DNase treatment of asthmatic mice improves lung resistance and decreases oxidative stress in the lungs, providing a potential antioxidant effect on asthma (65, 66). Accordingly, the combined use of recombinant human DNase therapy together with the current treatments (such as inhaled glucocorticoids) for severe acute asthma may prove effective in decreasing sputum viscosity, as it has been shown in specific case reports (67, 68).

## Chronic Obstructive Pulmonary Disease

Chronic obstructive pulmonary disease (COPD) is a progressive disorder of the airways characterized by persistent neutrophilic inflammation (69, 70). The disease develops following long-term exposure to external stresses, such as inhaled tobacco smoke (71–73). COPD patients are affected by recurrent bacterial and respiratory viral infections, which represent the main causes of exacerbations in these subjects. Exacerbations are associated with increased upper and lower airway and systemic inflammation (74). Patients with severe COPD present large amounts of airway neutrophils when stable, and these numbers further increase during exacerbations, which may be due to the high expression of neutrophil chemokines and chemokine receptors in airway mucosa (75). Furthermore, NE is expressed in the airway mucosa of COPD patients during severe exacerbations (75) and has a proinflammatory role by inducing the secretion of IL-8 in COPD (76). It is noteworthy that IL-8 is a potent NET inducer (3, 77). These features make COPD lungs more likely to be filled by NETs. Indeed, confocal microscopy analysis has shown that sputum from exacerbated COPD patients presents extracellular DNA, frequently entangled with bacteria (78), characterizing NETs. Moreover, NETs are present not only during COPD acute exacerbations, but also in the lungs of patients with stable disease (79–81). There is a clear correlation between the abundance of NETs in the sputum of COPD patients and disease severity – over 90% of exacerbated COPD subjects presented large amounts of NETs in their sputum compared to 45% of stable COPD subjects. In addition, the very large quantities of NETs directly correlate with the severity of airflow limitation in these patients (79). Why NETs are produced in excess in COPD and what would be the trigger for NET release are unsolved issues. Under physiological conditions, NETs would be degraded by endogenous nucleases and cleared by alveolar macrophages (82). However, COPD subjects present lower numbers of alveolar macrophages (81) and these macrophages are defective in phagocytosis (83), which may explain the persistence of NETs in the airways. Nonetheless, a recent interesting study has shown that the outcome of the interaction of macrophages and netting neutrophils depend on macrophage phenotype. M2 macrophages in contact with netting neutrophils helped to perpetuate an inflammatory response, while M1 macrophages initially released extracellular DNA and thereafter degraded DNA in a caspase-activated DNase-dependent manner (84). These findings highlight a phenotype-dependent mechanism of macrophage regulation of NET release, which reinforce the argument that a prolonged exposure to NETs may favor the development of autoimmunity. The exact role of NETs in COPD pathogenesis is uncertain, but the need for developing novel diagnostic and therapeutic strategies is clear. The treatment for COPD is very difficult, as anti-inflammatory drugs are ineffective. The most successful current treatment for COPD is long-acting bronchodilators, but no therapy reduces the progression or inhibits the inflammation (85). As NETs were implicated in disease worsening, selective inhibitors of NET formation or NET-associated proteins (such as NE, MPO, histones) may prove valuable in improving the clinical picture of the disease.

## Tuberculosis

Tuberculosis (TB) remains a major health problem for humankind. Annually, there are approximately nine million new cases and 1.5 million deaths caused by the disease (86). This chronic bacterial infection is caused by *Mycobacterium tuberculosis* and affects the lungs, promoting huge morbidity and mortality rates (86, 87). *M. tuberculosis* is usually transmitted by tiny droplets from cough or sneeze of an infected subject. Once in the lungs, the bacilli is phagocytosed and killed by alveolar macrophages. However, *M. tuberculosis* developed strategies to survive inside the macrophages. Therefore, the infection develops as a latent infection, inducing granuloma formation in the lung parenchyma. Consequently, the subject remains healthy while harboring dormant bacteria (87, 88). The key factor for the maintenance of latent TB infection is the equilibrium between the bacteria and the host immune response. TB reactivation is achieved when the immune response decreases and cannot restraint bacteria growth, inducing cell death and an increase in granulomatous lesions, as a result of inflammatory cell recruitment (88). Clinical symptoms of TB are caused by a severe impairment of lung function and by substantial morphological alterations in the lung parenchyma (87).

Although macrophages are generally viewed as the main cells involved in harboring *M. tuberculosis*, a growing body of evidence shows that neutrophils are rapidly recruited to infected lungs and can serve as bacterial reservoirs. Additionally, neutrophils were identified as the main immune cell type in sputum and BAL from active TB patients (89). Furthermore, human neutrophils are able to phagocytose *M. tuberculosis in vitro*, but fail to kill the bacilli (90). Neutrophils have been assigned to play both protective and pathological roles during active TB (91–93). As a part of their role in TB pathogenesis, neutrophils have been shown to release NETs coated with NE and histones when stimulated by two genotypes of *M. tuberculosis* (H37Rv and *M. canetti*). NETs were able to trap mycobacteria but not to kill them (94). This lack of killing ability of NETs may favor lung destruction in active TB. Another study has found matrix metalloproteinase-8 (MMP-8) expressed on NET fibers induced by *M. tuberculosis in vitro*. In addition, induced sputum from TB patients had increased amounts of NETs compared to healthy subjects and MMP-8 secretion correlated to lung tissue destruction in these patients (95). The effect of *M. tuberculosis* on NET induction might be mediated by the early secretory antigen-6 (ESAT-6), a protein secreted by *M. tuberculosis*, responsible for the escape of mycobacteria from phagosome to cytoplasm of cells (96), as ESAT-6 induces the production of NETs colocalized with MPO (97). ESAT-6 is also secreted in large quantities in the extracellular space and therefore can interact with immune cells to stimulate them and facilitate the maintenance of chronic inflammation in the lungs of TB patients (98). Importantly, neutrophils release high levels of calprotectin (S100A8/A9) within lung granulomas of patients with active TB (99), which are constituents of NETs (100). The release of calprotectin in TB could be related to NET formation, as neutrophil cytoplasmic proteins can attach to DNA fibers before being released. Urban and coworkers have shown that calprotectin can be released from neutrophils in two ways: bound to NETs and unbound (100). This could be the case for calprotectin release in the lungs of TB subjects; however whether *M. tuberculosis* induces the formation of NETs expressing calprotectin remains to be determined.

Tuberculosis is a curable disease, although the treatment is difficult, since it can take several months (6–9 months) and has different drug regimens. Currently, the first line anti-TB drugs include isoniazid, rifampin, ethambutol, and pyrazinamide, among others when necessary, according to CDC (Centers for Disease Control and Prevention – http://www.cdc.gov/tb/topic/treatment/). Moreover, new therapies aiming to improve the treatment outcomes, shorten the duration of treatment, and reduce lung pathology in TB patients were described (101). However, no therapeutic approach aimed to specifically regulate the deleterious effects of NETs in TB lungs was reported.

## Bacterial Pneumonia

The most common type of bacterial pneumonia is community-acquired pneumonia (CAP). CAP remains a burden worldwide, being responsible for approximately 3.5 million deaths annually (102). A total of 20–60% of CAP patients require hospitalization due to disease severity, including children under age 5 years (102, 103). The etiology of CAP is variable, depending partly on the diagnostic tools used in the population studied. Among all bacteria, *Streptococcus pneumoniae* (*S. pneumoniae*) is the most frequently identified cause of CAP, with high morbidity and mortality rates, but *H. influenzae* is also an important etiologic agent of CAP (102, 104).

Once bacterial infection is established in the lungs, neutrophils are massively recruited to the infection site, inducing a prominent inflammatory response. The clinical outcome in CAP depends on the balance between the inflammatory response and pathogen clearance (102). In this sense, neutrophils actively producing NETs during CAP might lead to potential collateral damage to the lungs. Indeed, three different strains of *S. pneumoniae* (serotypes 3, 4, and 19F) were able to induce pulmonary NET formation in mice, which correlated with the histopathologic severity. In addition, the pneumococcal capsule directly contributes to excessive NET release that paralleled with pneumonia severity in mice (105). The mechanism of NET induction by *S. pneumoniae* seems to be mediated by the pneumococcal protein α-enolase, which binds to myoblast antigen 24.1D5 on neutrophil surface and stimulates NET generation (106). However, *S. pneumoniae* appears to have evolved strategies to counteract NET-mediated killing. In an elegant study, Beiter and colleagues have demonstrated that *S. pneumoniae* expresses EndA, a membrane-localized endonuclease able to degrade NETs *in vitro* and to promote spreading of bacteria from the upper airways to the lungs and from the lungs to the bloodstream of mice. Additionally, mutant bacteria lacking EndA infect the upper airways but fail to disseminate to the lungs and bloodstream (107). Moreover, EndA is secreted into the culture medium during pneumococcal cell growth and rapidly dismantle DNA in NETs, being required for full virulence of *S. pneumoniae* during lung infection (108). Corroborating with these studies, streptococcal endonuclease has been previously implicated in disease progression (109). Besides EndA, streptococcal cells hold other important mechanisms to protect them from NET trapping and killing, such as a positive charge on their surfaces as a result of capsule expression and lipoteichoic acid d-alanylation (110). Thus, it seems that NETs released during *S. pneumoniae* infection function only to damage lung tissue, instead of having a bactericidal activity. The evidence that NETs released in response to bacterial infections can trap and inactivate viruses (8, 111) points out the utmost importance of NETs during co-infections *in vivo*. On the other hand, secondary pneumococcal infection following primary influenza intensified NET formation, but NETs did not show any bactericidal activity, only worsening lung pathogenesis (112). Altogether, these findings suggest that the nature of NET trigger is fundamental to the clearance of subsequent infections.

Non-typeable *H. influenzae* (which lacks a capsule) is an important cause of pneumonia, mainly in subjects with chronic bronchitis and COPD (113), and the persistence of NETs could worsen lung inflammation in these subjects. Viable and heat-killed *H. influenzae* induces NET release *in vitro*, in a mechanism possibly mediated by lipooligosaccharide binding to TLR-4 and Myeloid Differentiation Primary Response (MyD)-88, an adaptor protein necessary to TLR-4 signaling. Interestingly, bacteria are not killed by NET proteins and survive within NETs (114). Accordingly, it has been recently demonstrated that these bacteria evolved to express specific molecules, peroxiredoxin–glutaredoxin and catalase, which allow them to resist to host oxidants and to survive within NETs *in vivo* (115). In addition, non-typeable *H. influenzae* populations survive in biofilm communities in the airway surface, and NETs constitute an integral part of these biofilms (116). Astoundingly, it has been reported a fatal case of non-typeable *H. influenzae* infection with severe pneumonia and bacteremia in an adult found to have large amounts of NETs expressing NE and histone H3 in his sputum (117). This case highlights the association between excessive NET generation and severe respiratory infection and sepsis. More recently, it has been shown that besides NETs, non-typeable *H. influenzae* is also able to induce macrophage extracellular traps (METs) expressing MMP-12 (118). MMP-12 has been implicated as a key factor for protease imbalance and emphysema. Therefore, the release of METs together with NETs may have a detrimental role during emphysema, pneumonia, and COPD. Importantly, DNase was effective to dismantle non-typeable *H. influenzae*-induced MET and NET formation (118), which could be used as a short-term adjunctive therapy to avoid the injurious effects of these extracellular traps and associated proteases during pneumonia and other lung diseases.

## Respiratory Syncytial Virus Bronchiolitis

Respiratory Syncytial Virus (RSV) is the leading cause of acute bronchiolitis in children under age 2 years (119). Throughout the winter, RSV causes a significant number of hospitalizations, resulting in a huge burden to communities worldwide (119, 120). Due to the high infectivity of RSV, almost 70% of all children are infected with the virus during the first year of life, and by age 3, practically all children will have experienced at least one infection with this virus (121, 122). The clinical symptoms of RSV bronchiolitis include labored breathing, coughing, and wheezing (123). Microscopically, there is a massive neutrophil recruitment to the airways of infected children – these cells comprise for approximately 80% of infiltrated cells (124–127). Once in the airways, RSV is able to activate neutrophils, inducing degranulation and IL-8 secretion (128), and also to inhibit neutrophil apoptosis, through phosphoinositide 3-kinase (PI3K) and nuclear factor-κB (NF-κB)-dependent mechanisms (129). This body of evidence suggests that neutrophils may play a significant role in disease pathogenesis.

Aside from the mechanisms mentioned above, we have recently demonstrated that RSV particles and one of its membrane-bound glycoproteins are capable of inducing NET formation by human neutrophils (130). RSV Fusion protein mediates the fusion of virus with the host cell and it is essential for viral replication both *in vivo* and *in vitro* (131), being considered the primary target for vaccine and antiviral drug development. RSV F protein induces the release of NETs coated with MPO and NE through Toll-like receptor (TLR)-4 activation. Moreover, F protein stimulates ROS generation and MAPK phosphorylation, and these signaling pathways are necessary to F protein-induced NET formation (130). Data in the literature regarding the role of NETs in viral diseases are conflicting (132). We hypothesized that the excessive production of NETs could fill the lungs and impair lung function, worsening inflammation in young children and babies affected by RSV infection. Indeed, analysis of bronchoalveolar fluid cytology samples from children with severe RSV lower respiratory tract infection revealed the presence of NETs expressing NE and citrullinated histone 3 (citH3) (133). Furthermore, the infection of calves with bovine RSV induced an extensive release of NETs colocalized with dense cellular plugs containing shed epithelial cells and large amounts of neutrophils, which obstructed the airways (133). These recent studies indicate that NETs contribute to the airway obstruction and immunopathology observed in children and animals infected with RSV.

Despite extensive research efforts, there is no RSV vaccine currently available. Nevertheless, monoclonal antibodies targeting the RSV fusion protein have been developed and they passively protect against RSV challenge in an animal model and reduce the severity of infection in premature and newborn babies (134, 135). However, the humanized monoclonal antibody against RSV F protein is only used in high-risk groups, such as preterm infants and those suffering from cardiovascular diseases or immunosuppression (134). In addition, ribavirin is an antiviral drug used to treat severe RSV bronchiolitis due to its anti-replicative activity, but it presents a high cost and is administered only to high-risk infants (136). Moreover, the use of recombinant human DNase in the management of severe RSV bronchiolitis has been previously reported. The administration of nebulized DNase to young babies with complicated bronchiolitis was able to immediately improve the clinical signs and chest radiograph, and even led to the resolution of atelectasis (137, 138). In contrast, in infants with mild RSV bronchiolitis, recombinant DNase therapy did not reduce the length of hospital stay or the duration of supplemental oxygen (139). Thus, DNase seems to be a useful therapeutic option in the treatment of infants who develop atelectasis due to severe RSV bronchiolitis.

## Influenza Virus Infection

Influenza A virus is responsible for regular outbreaks, whose severity may vary among the population. While the influenza pandemic that started with the Spanish flu in 1918 killed approximately 50 million people worldwide, the pandemic influenza A H1N1 2009 virus has affected more than 214 countries and caused nearly 18,449 deaths (140, 141). The clinical features of influenza infection include fever and upper respiratory symptoms, such as cough, runny nose, and sore throat (141). To date, there is little information about clinical complications of influenza A infection, but they appear to be similar to those of seasonal influenza, including sinusitis, otitis media, pneumonia, bronchiolitis, seizures, toxic shock syndrome, and secondary bacterial pneumonia with or without sepsis. Among subjects with high risk for complications are those at extremes of age and those with pre-existing medical conditions (141).

The characteristic feature of acute lung inflammation following influenza virus infection is the excessive infiltration of neutrophils in the lungs (142, 143), and CXCR2 seems to be the major receptor mediating neutrophil recruitment during this infection (144). Neutrophils have been demonstrated to play both protective and detrimental roles during influenza virus infection (143, 145, 146). Among the harmful roles played by neutrophils is the excessive production of NETs in the lungs of animals infected with influenza A H1N1 virus. NETs expressing histones and MMP-9 were found entangled with alveoli, causing increased alveolar capillary damage and obstruction of the small airways, thus confirming the link of these DNA lattices with lung damage (146). Furthermore, NET formation stimulated by influenza A infection is dependent on histone deimination by PAD4 (147). In addition, NET release induced by influenza virus is potentiated by the cathelicidin LL-37 (148), which has been shown to facilitate the formation of NETs (149). Paradoxically, the antimicrobial protein expressed on NETs, α-defensin-1, is able to directly inhibit influenza replication through the inhibition of protein kinase C (PKC) in infected cells (150); however the expression of α-defensins on NETs induced by this virus has yet to be demonstrated. The expression of α-defensins on NETs could inactivate the virions sequestered in NET fibers and consequently prevent them from reaching the target cells in the lungs. Thus, although antimicrobial proteins expressed on NETs have the ability to inactivate the virus and to prevent spreading, they are also able to inflict damage to host cells and tissues due to their cytotoxic properties.

Currently, influenza treatment relies on the administration of two groups of antiviral drugs, the adamantanes and neuraminidase inhibitors. Zanamivir and oseltamivir are neuraminidase inhibitors active against both influenza A and B, and are approved for the prevention and treatment of influenza in the United States. Supportive care of uncomplicated cases of influenza includes administration of fluids and rest (141). To date, there is no study describing the effect of DNase treatment on the outcome of influenza infection in animal models.

## Transfusion-Related Acute Lung Injury

Transfusion-related acute lung injury (TRALI) is a serious complication of blood transfusion (whole blood or blood components) that develops within 6 h of transfusion and is characterized by hypoxemia, respiratory distress, and pulmonary infiltrates (151, 152). Currently, TRALI is the most important cause of transfusion-related morbidity and mortality (152). Histological analysis revealed lung edema, capillary leucostasis, and massive neutrophil infiltration (153). TRALI development requires the presence of antileukocyte antibodies in the transfused product, and antineutrophil antibodies have been linked to the most severe cases of TRALI (154). These antibodies activate recipient’s neutrophils, inducing their sequestration in the pulmonary capillaries and consequently tissue injury (155).

In an elegant study, Thomas and coworkers have found NET biomarkers (DNA, nucleosomes and MPO) in the serum of patients with documented TRALI (156). In addition, in a fatal case of TRALI neutrophils with decondensed nuclei were detected in lung vessels together with abundant extracellular histones and MPO (157). In a mouse model of TRALI, DNA streaks colocalizing with citrullinated histone H3 were found in alveoli outside blood vessels (156). Moreover, platelets also accumulate in the lungs of mice with TRALI, being required for injury development (158). In this model, platelets were shown to induce NET formation during TRALI (157). As a vicious cycle, histones expressed on NETs may activate platelets (159), which in turn induce further NET release, promoting coagulation and thrombi formation in the lungs. Accordingly, the pretreatment of mice with a histone-blocking antibody decreased lung edema, lung vascular permeability, and even mortality. This treatment also reduced NET generation detected in plasma, indicating that extracellular histones may help to spread NETs in the body (157). Furthermore, intranasal administration of DNase provided several benefits to mice undergoing TRALI, such as improvement of blood oxygenation, reduction in lung edema and vascular permeability, impairment of NET formation, and platelet sequestration in the lungs (156, 157). These studies support the argument that NETs are formed and play a critical role in the pathogenesis of TRALI and may be a promising target for therapeutic approaches.

## Mechanical Ventilation

Mechanical ventilation is a supportive intervention and a key feature of intensive care for patients with acute respiratory failure, including those with severe RSV bronchiolitis, pneumonia, or influenza infection (160, 161). However, it can be potentially injurious to the ventilated lung, inducing the so-called ventilator-associated lung injury (VALI), which contributes to morbidity and mortality in those patients (162). Furthermore, animal models of acute lung injury have been developed and characterize an experimental insult to a normal lung and therefore were named ventilator-induced lung injury (VILI) (162).

Neutrophils have been implicated as central cells in the pathogenesis of both VALI and VILI. It has been described that the early phase of VILI involves the release of several pro-inflammatory cytokines and chemokines, whereas the late phase is characterized by the infiltration of a lung-marginated neutrophil pool (163, 164). However, more recently Choudhury and coworkers demonstrated that injurious mechanical ventilation induced a prominent neutrophil recruitment to the lung at the very early stage of VILI, before the development of physiological signs of lung injury. The infiltration of neutrophils in the course of VILI was dependent on L-selectin engagement but independent of CD18 (165), indicating that immune mechanisms mediate neutrophil recruitment and activation during mechanical ventilation. Moreover, lung-derived soluble mediators appear to have a pathogenic role in an isolated perfused lung model of VILI (166). In line with this evidence, the chemokine receptor CXCR2 and its ligands, CXCL1 (KC) and CXCL2/3 (MIP-2), were shown to play a significant role in mediating neutrophil recruitment and promoting lung inflammation in VILI (167). Accordingly, short periods of mechanical ventilation in preterm infants induce an overproduction of the pro-inflammatory cytokines TNF and IL-1β, neutrophil chemokines IL-8 and MCP-1, and MMP-9 (168, 169). These inflammatory mediators may work together to induce a massive neutrophil infiltration to ventilated lungs and to stimulate NET release in response to mechanical ventilation in those patients. So far, IL-8, TNF, and IL-1β were shown to promote NET release in different experimental settings (10, 170, 171). In fact, excessive NET formation has been recently implicated in the pathogenesis of VILI. A double-hit model of intratracheal LPS challenge followed by high tidal mechanical ventilation induced a prominent lung injury in mice, with high amounts of NETs, decreased lung compliance and release of pro-inflammatory cytokines (172). The mechanism of NET formation during VILI seems to rely on the simultaneous engagement of G protein-coupled receptors (GPCR) and Mac-1 (CD11b), by the platelet-derived CCL5/CXCL4 heterodimer and a β2-integrin ligand, respectively (173). Surprisingly, these two studies showed opposing results regarding the role of NETs during VILI. Rossaint and coworkers found that DNase treatment of mice after induction of VILI was protective, as treated mice showed an improved gas exchange and reduced NET markers in the blood; whereas Yildiz and colleagues did not find a significant impact of DNase treatment on lung injury induced by VILI. There is at least one possible explanation for these differences: in the study of Yildiz and colleagues, the lungs of mice were already filled with neutrophils at the early stage of VILI due to LPS instillation, which could not be counteracted by DNase. Whereas in the study of Rossaint and coworkers, neutrophils infiltrated the lungs in the course of VILI, in this case a sterile inflammation. Although the outcome of DNase treatment in VILI is an issue for debate, there is no doubt that excessive NET formation accounts for the pathogenesis of acute lung injury.

## Other Pulmonary Diseases and NETs

Besides the pulmonary diseases aforementioned, there are other disorders or syndromes affecting the lungs, in which NETs may play harmful roles as well.

Acute lung injury following severe sepsis is a common clinical consequence with significant morbidity and mortality rates (174), as the lung is the most sensitive target organ during systemic inflammation (175). Czaikoski and collaborators have recently shown that NETs are produced systemically in mice with cecal ligation and puncture (CLP) model of sepsis. The excessive release of NETs was directly correlated to heart, liver, and lung injury, as rhDNase plus antibiotics treatment of septic mice drastically decreased organ damage (176). Additionally to extracellular DNA measurement, NETs were observed in alveolar spaces and pulmonary capillaries of septic mice (177). Furthermore, higher concentrations of cell-free NETs were present in the serum of septic patients who developed severe acute respiratory distress syndrome (ARDS) compared to healthy controls (176), extending the experimental observations in mice to the clinical setting. Mechanistically, platelet TLR-4 is essential for NET induction within hepatic sinusoids and pulmonary capillaries of septic mice (178). Interestingly, NETs retained their integrity under flow conditions and were able to trap bacteria in septic blood. Therefore, platelets may serve as a platform for neutrophil activation and NET production, which can trap and kill pathogens but also induce disseminated organ injury during severe sepsis (178).

Another lung disorder featuring neutrophil-induced injury is interstitial lung disease (ILD). Actually, ILD are a group of diffuse parenchymal lung disorders characterized by pulmonary fibrosis. ILD can be frequently associated with a specific environmental exposure or an underlying connective tissue disease (179). Activated neutrophils were found increased in BAL from patients with idiopathic pulmonary fibrosis and were associated with early mortality (180). Interestingly, patients with ILD complications due to autoimmunity showed elevated levels of circulating cell-free NETs and plasma LL-37 (a NET component), together with a decreased DNase activity (181), suggesting that the prolonged exposure to NETs is involved in the pathogenesis of ILD. *In vitro*, NETs have been demonstrated to promote the activation of lung fibroblasts and differentiation into myofibroblast phenotype. Moreover, these fibrotic effects were significantly decreased after degradation of NETs with DNase (182). Consistently, these findings were supported by the detection of NETs in close proximity to alpha-smooth muscle actin-expressing fibroblasts in biopsies from patients with fibrotic ILD (182). This effect is very likely to be mediated by NE, since NE directed both lung fibroblast proliferation and myofibroblast differentiation *in vitro* (183). In addition, a NE inhibitor attenuated pulmonary fibrosis induced by bleomycin in mice via inhibition of TGF-β1 and inflammatory cell recruitment to the lungs (184). Altogether, these studies point to a key role of NETs in the development of ILD of different etiologies.

## Conclusion

**Figure 1:**
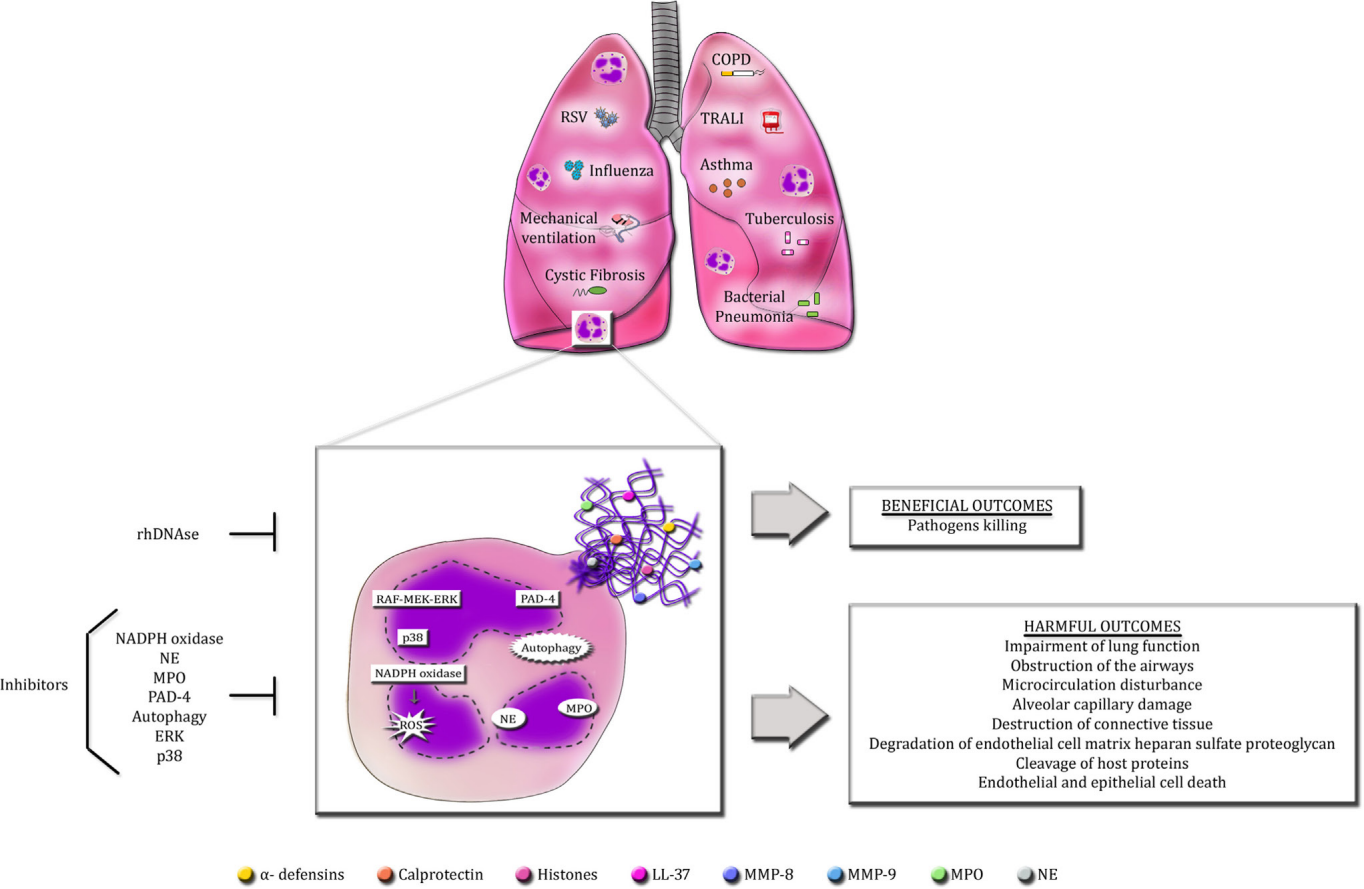
**Overview of the beneficial and detrimental roles of NETs in pulmonary diseases**. Infectious and non-infectious pulmonary diseases cause the massive infiltration of neutrophils into the lungs. Activated neutrophils release an excess of NETs in the airways. The production of NETs requires the activation of specific signaling pathways described so far, such as raf-MEK-ERK and p38 MAPK, PAD-4, autophagy and NADPH oxidase-induced ROS generation. Additionally, NE and MPO also regulate NET formation. Accordingly, selective inhibitors of these signaling pathways are able to abolish or decrease NET release. The primary goal of NETs is to protect the host from invading microorganisms through their sticky nature and the high concentrations of antimicrobial proteins. However, these characteristics make NETs potentially detrimental to host cells and tissues. Excessive NET formation enhances mucus viscosity, filling the lungs, and impairing lung function. NET proteins are highly cytotoxic and can induce endothelial and epithelial cell death and cause the disruption of host proteins and cellular matrix.

Neutrophil extracellular traps formation by activated neutrophils has a crucial role in host defense against microorganisms, as deficiency in NET release or dismantling NET backbone by bacterial DNases render the host susceptible to disseminated and lethal infections (107, 185). Moreover, aggregated NETs have been shown to limit sterile inflammation by degrading cytokines and chemokines via serine proteases (186). However, an excess or persistence of NET release is potentially injurious to host organs and cells, leading to worsening or perpetuation of many diseases. The pathogenic effects of excessive NET production is especially important in pulmonary diseases due to lung architecture itself, which may favor the spreading of DNA fibers, consequently enhancing tissue damage and impairing lung function (Figure 1). The mechanisms underlying NET production and the boundaries between the beneficial and detrimental effects of NETs during disease state are still to be unveiled. To date, recombinant human DNase is the only treatment targeting NETs approved for a small number of pulmonary disorders. Nevertheless, a long-term DNase therapy presents side effects to patients. Hence, the quest for an ideal therapy targeting NETs and its associated proteins continues to be a challenge for scientists around the globe.

## Author Contributions

All authors listed, have made substantial, direct and intellectual contribution to the work, and approved it for publication.

## Conflict of Interest Statement

The authors declare that the research was conducted in the absence of any commercial or financial relationships that could be construed as a potential conflict of interest.
